# Mitochondrial dysfunction and metabolic reprogramming induce macrophage pro-inflammatory phenotype switch and atherosclerosis progression in aging

**DOI:** 10.3389/fimmu.2024.1410832

**Published:** 2024-06-21

**Authors:** Aleksandr E. Vendrov, Andrey Lozhkin, Takayuki Hayami, Julia Levin, Jamille Silveira Fernandes Chamon, Ahmed Abdel-Latif, Marschall S. Runge, Nageswara R. Madamanchi

**Affiliations:** ^1^ Frankel Cardiovascular Center, Division of Cardiovascular Medicine, Department of Internal Medicine, University of Michigan, Ann Arbor, MI, United States; ^2^ Department of Internal Medicine - Cardiology, Ann Arbor VA Healthcare System, Ann Arbor, MI, United States

**Keywords:** NOX4 NADPH oxidase, mitochondrial dysfunction, macrophages, atherosclerosis, aging

## Abstract

**Introduction:**

Aging increases the risk of atherosclerotic vascular disease and its complications. Macrophages are pivotal in the pathogenesis of vascular aging, driving inflammation and atherosclerosis progression. NOX4 (NADPH oxidase 4) expression increases with age, correlating with mitochondrial dysfunction, inflammation, and atherosclerosis. We hypothesized that the NOX4-dependent mitochondrial oxidative stress promotes aging-associated atherosclerosis progression by causing metabolic dysfunction and inflammatory phenotype switch in macrophages.

**Methods:**

We studied atherosclerotic lesion morphology and macrophage phenotype in young (5-month-old) and aged (16-month-old) *Nox4* ^-/-^/*Apoe* ^-/-^ and *Apoe* ^-/-^ mice fed Western diet.

**Results:**

Young *Nox4^-/-^/Apoe^-/-^* and *Apoe^-/-^* mice had comparable aortic and brachiocephalic artery atherosclerotic lesion cross-sectional areas. Aged mice showed significantly increased lesion area compared with young mice. Aged *Nox4^-/-^/Apoe^-/-^* had significantly lower lesion areas than Apoe-/- mice. Compared with Apoe-/- mice, atherosclerotic lesions in aged *Nox4^-/-^/Apoe^-/-^* showed reduced cellular and mitochondrial ROS and oxidative DNA damage, lower necrotic core area, higher collagen content, and decreased inflammatory cytokine expression. Immunofluorescence and flow cytometry analysis revealed that aged *Apoe^-/-^* mice had a higher percentage of classically activated pro-inflammatory macrophages (CD38^+^CD80^+^) in the lesions. Aged *Nox4^-/-^/Apoe^-/-^* mice had a significantly higher proportion of alternatively activated pro-resolving macrophages (EGR2^+^/CD163^+^CD206^+^) in the lesions, with an increased CD38^+^/EGR2^+^ cell ratio compared with *Apoe^-/-^* mice. Mitochondrial respiration assessment revealed impaired oxidative phosphorylation and increased glycolytic ATP production in macrophages from aged *Apoe^-/-^* mice. In contrast, macrophages from *Nox4^-/-^/Apoe^-/-^* mice were less glycolytic and more aerobic, with preserved basal and maximal respiration and mitochondrial ATP production. Macrophages from *Nox4^-/-^/Apoe^-/-^* mice also had lower mitochondrial ROS levels and reduced IL1β secretion; flow cytometry analysis showed fewer CD38+ cells after IFNγ+LPS treatment and more EGR2^+^ cells after IL4 treatment than in *Apoe^-/-^* macrophages. In aged *Apoe^-/-^* mice, inhibition of NOX4 activity using GKT137831 significantly reduced macrophage mitochondrial ROS and improved mitochondrial function, resulting in decreased CD68^+^CD80^+^ and increased CD163^+^CD206^+^ lesion macrophage proportion and attenuated atherosclerosis.

**Discussion:**

Our findings suggest that increased NOX4 in aging drives macrophage mitochondrial dysfunction, glycolytic metabolic switch, and pro-inflammatory phenotype, advancing atherosclerosis. Inhibiting NOX4 or mitochondrial dysfunction could alleviate vascular inflammation and atherosclerosis, preserving plaque integrity.

## Introduction

Aging is a major risk factor for cardiovascular diseases, including atherosclerosis. The prevalence of coronary artery disease (CAD) increases with age, reaching 22% in males and 12% in females aged 60–79, compared with 7% in 40–59-year-olds (1). This trend is mirrored in the average age at first myocardial infarction, which stands at 65 years for males and 72 years for females (1). Atherosclerosis progression is associated with aging-related structural and functional vascular changes, including wall remodeling and decreased compliance, vascular cells oxidative stress, phenotype changes and dysfunction, and development of chronic unresolved inflammation (2, 3). Recent clinical studies suggest that inflammation plays a significant role in vascular aging and atherosclerosis (4, 5). However, not all anti-inflammatory therapies for CAD showed efficacy, especially those that target immune cells (6), suggesting a complex interplay between inflammation and clinical outcomes.

Immune cells, including monocytes, are recruited to the vascular wall in atherogenesis and play a critical role in sustaining oxidative stress, inflammation, and extracellular matrix degradation. Atherosclerotic lesion macrophages can maintain several phenotypes, including classically activated (M1 or M[IFNγ+LPS]) pro-inflammatory macrophages and alternatively activated (M2 or M[IL4]) pro-resolving macrophages (7). Macrophage metabolic reprogramming, with pro-inflammatory cells relying on glycolysis and pro-resolving cells on oxidative phosphorylation for energy production (8), is closely related to the changes in atherosclerotic plaque environment and morphology (9). Nevertheless, the mechanisms of metabolic reprogramming of macrophages in atherosclerosis and its effects on plaque morphology are incompletely understood.

Mitochondrial dysfunction in macrophages in aging results in reduced ATP production, elevated reactive oxygen species (ROS) generation, and compromised mitochondrial quality control, features that are intricately linked to the shift in metabolism from oxidative phosphorylation to glycolysis and pro-inflammatory phenotype (9, 10). Consequently, aging-associated atherosclerotic plaque mitochondrial oxidative stress and dysfunction result in increased lesion volume and vulnerable plaque features (11, 12). Expression of mitochondria-localized NOX4 NADPH oxidase is increased with age in human and mouse vasculature and is associated with increased oxidative stress, vascular inflammation, aortic stiffness, and atherosclerotic lesion size and severity (13–15). Similarly, increased NOX4 expression in atherosclerotic plaque was associated with plaque instability and rupture (16), while direct inhibition, genetic downregulation of NOX4, or blockade of NOX4-dependent signaling pathways inhibited atherogenesis (14, 15, 17, 18). In human coronary atherosclerotic lesions increased NOX4 expression was observed in nonphagocytic vascular cells, contributing to increased ROS levels (19), while increased NOX4-derived ROS in human monocytes was associated with higher metabolic priming, vascular recruitment and atherosclerosis progression (20, 21).

Targeting NOX4-dependent mitochondrial ROS holds promise in atherosclerosis management (12, 14). However, the precise mechanisms of mitochondrial dysfunction in aging-associated atherosclerosis, its impact on plaque progression and phenotype, and therapeutic potential are not fully elucidated. Here, we tested the hypothesis that mitochondrial oxidative stress associated with increased NOX4 levels in aging results in metabolic priming in monocytes/macrophages to pro-inflammatory phenotype switch, fostering atherosclerotic lesion progression. We used *Apoe^-/-^* mice as they have high cholesterol levels when fed a Western diet, leading to human-like atherosclerosis progression with similar lesion cellular composition, a prominent inflammatory profile, and aging-related phenotype useful for aging studies (22–24). Effects of aging were examined in 16-month-old mice, which represent the age equivalent of humans with exponentially increasing coronary heart disease incidence, making them a useful model to study the pathogenesis of atherosclerosis (1, 22). Using aged *Nox4*-deficient *Apoe^-/-^* mice, mice we showed that reduced mitochondrial ROS in macrophages preserves mitochondrial function and is associated with pro-resolving phenotype attenuating atherosclerotic disease. We recapitulated our findings by inhibiting NOX4 activity in aged *Apoe* ^-/-^ mice.

## Materials and methods

### Animals

All animal procedures were performed in compliance with the protocols approved by University of Michigan Institutional Animal Care and Use Committee in accordance with NIH OLAW policy and Guide for the Care and Use of Laboratory Animals, 8^th^ ed. Male wild-type C57BL/6J, *Nox4* ^-/-^ (B6.129-*Nox4^tm1Kkr^*/J), and *Apoe* ^-/-^ (B6.129P2-*Apoe^tm1Unc^*/J) mice were purchased from the Jackson Laboratory (Bar Harbor, ME). Littermate male mice were used in all experiments. Mice were housed in ventilated cages at 22°C with 12-hour light/dark cycle and free access to food and water. For atherosclerosis analysis *Apoe* ^-/-^ and *Nox4* ^-/-^/*Apoe* ^-/-^ mice were fed standard rodent chow until 8-weeks-old, then fed Western type diet (TD.88137; Envigo, Madison, WI) for 12 weeks. For assessment of atherosclerosis in aging, mice were kept on standard rodent chow until 13-month-old, then fed Western diet for additional 12 weeks.

### Cell culture

Monocytes were isolated from *Apoe* ^-/-^ and *Nox4* ^-/-^/*Apoe* ^-/-^ mice as described (23). Briefly, mice were euthanized with inhaled isoflurane overdose, the spleens were dissected and mechanically dispersed. Cell suspension was passed through a 70 µm cell strainer (Corning, Corning, NY) and incubated in RBC lysis buffer (15.5 mM NH4Cl, 1 mM KHCO3, 10 µM EDTA, pH7.3). Cells were washed and cultured in RPMI1640 medium supplemented with 10% fetal bovine serum, 10 ng/mL M-CSF and antibiotic/antimycotic solution (Thermo Fisher; Waltham, MA) in a 5% CO_2_ incubator at 37°C. Cells were treated with either 20 ng/mL IFNγ (PeproTech; Cranbury, NJ) and 10 ng/mL LPS (Sigma-Aldrich; St. Louis, MO), or 20 ng/mL IL4 (PeproTech) for 24 hours to induce M[IFNγ+LPS] (M1) or M[IL4] (M2) phenotypes, respectively. Cells treated with vehicle were used as M0 controls. Alternatively, cells were pretreated with 10 μM GKT137831 (Cayman Chemical; Ann Arbor, MI) for 30 minutes prior other treatments.

### Histology and immunostaining

Mice were euthanized with inhaled isoflurane overdose and aortas were dissected, fixed and atherosclerotic lesion area determined as previously described (24). Longitudinally opened aortas were stained with 1% oil red O and 0.1% toluidine blue (Sigma). Digital images of stained aortas were analyzed with NIH ImageJ 1.54f (Bethesda, MD). Mouse hearts and proximal brachiocephalic artery were dissected, embedded in O.C.T. compound (Sakura Finetek; Torrance, CA), and snap-frozen in liquid nitrogen. Transverse 10 μm serial sections of aortic root and brachiocephalic artery were collected and processed as described before (24).

Picrosirius red staining was performed with brachiocephalic artery cryosections as previously described (25). Briefly, sections were fixed in 3.7% paraformaldehyde stained using Picrosirius Red Stain Kit (Abcam; Waltham, MA). Images were acquired with Revolve R4 microscope (Echo; San Diego, CA) in brightfield mode and analyzed with NIH ImageJ software.

Immunofluorescence staining was performed as previously described (24, 26). Cryosections were fixed in acetone, permeabilized in 0.1% Triton X-100 and immunostaining was done using antibodies against 8-OHdG, NOX4, CCL2, IL1b, TOM20-AlexaFluor488 (Abcam), CD68 (Thermo Fisher); ACTA2-FITC (Sigma); CD11b (Abnova; Walnut, CA); IL6 (Cell Signaling Technology; Danvers, MA); ATP5G-AlexaFluor488, CD80-AlexaFluor488, CD68-Cy3, CD163-AlexaFluor594, CD206-AlexaFluor488 (Bioss). The goat anti-rabbit antibodies conjugated to AlexaFluor488 or AlexaFluor594, or rabbit anti-goat AlexaFluor594 secondary antibodies were used when appropriate. Sections were mounted with ProLong Gold reagent with DAPI (Thermo Fisher), fluorescence images were taken with Nikon Microphot-FX microscope and analyzed using NIH ImageJ.

### ROS detection

Brachiocephalic artery cryosections were analyzed immediately after collection. Cellular ROS levels were determined with dihydroethidium (Thermo Fisher) fluorescence and mitochondrial ROS with MitoSOX Red (Thermo Fisher) fluorescence as previously described (12, 14). Fluorescence images were taken with Nikon Microphot-FX microscope using 510 nm excitation/580 nm emission filters. Images were analyzed using NIH ImageJ software. Control sections incubated with PEG-SOD were used for background/autofluorescence correction.

AmplexRed assay (Thermo Fisher) was used to determine hydrogen peroxide release from freshly dissected aorta samples as previously described (27). Briefly, dissected aortas were gently minced and incubated in AmplexRed working solution for 30 minutes. Fluorescence was measured in sample aliquots using Spectramax iD5 Multi-Mode Microplate Reader (Molecular Devices; San Jose, CA) and the amount of H_2_O_2_ was determined using standard curve. Dry tissue weight was determined and used to normalize H_2_O_2_ levels.

Superoxide levels in cultured macrophages were determined with HPLC measurement of 2-hydroxyethidium levels as described previously (12) based on the method described by Zielonka et al. (28). Briefly, Samples were analyzed using Agilent 1100 HPLC system equipped with Kinetex 2.6 µm C18 100Å 100x4.6 mm LC column (Phenomenex; Torrance, CA). The amount of 2-hydroxyethidium was determined using oxyethidium (Noxygen Science Transfer & Diagnostics GmbH; Elzach, Germany) standard curve and normalized to the sample protein concentration measured with Pierce BCA Protein Assay Kit (Thermo Fisher).

Mitochondrial ROS in cultured macrophages were determined with MitoSOX Red (Thermo Fisher) fluorescence as described (14, 25). Briefly, cells were grown on tissue culture treated glass chamber slides (Corning Life Sciences; Tewksbury, MA), then, after treatment, cells were incubated with 10 µM MitoSOX Red at 37C° for 30 minutes and confocal images taken with Echo Revolve R4 microscope. Single cell images were analyzed with NIH ImageJ software. Control cells incubated with PEG-SOD were used for background/autofluorescence correction.

### Flow cytometry

Analysis of peripheral blood mononuclear cells was performed as described previously (29). Blood samples were collected in Lithium Heparin BD Vacutainers (BD Biosciences; Franklin Lakes, NJ). Red blood cells were lysed with RBC Lysis Buffer (Thermo Fisher). The remaining cells were passed through a 70 µm cell strainer (Corning) and washed with PBS. Cells were blocked with rat anti-mouse CD16/CD32 antibody mix (BD Biosciences), then stained with Zombie UV Viability Dye (BioLegend; San Diego, CA). CD11b-APC, LY6C-FITC (Miltenyi Biotech; Gaithersburg, MD), and CD115-AlexaFluor532 (Novus Biological; Centennial, CO) antibodies were used for staining. The samples were processed with MoFlo Astrios EQ Cell Sorter (Beckman Coulter; Brea, CA) and analyzed using FlowJo v10.8 (BD Biosciences). Peripheral blood mononuclear cells were identified as CD11b^+^CD115^+^LY6C^+^ fraction of all cells. Based on the LY6C fluorescence intensity LY6C^hi^ and LY6C^lo^ cells were identified (**Supplementary Figure 1**).

Analysis of aortic atherosclerotic lesions-derived single-cell suspension was done as reported previously (24). Briefly, dissected aortas were gently minced and dissociated in collagenase type I (400 U/mL), collagenase type XI (120 U/mL), hyaluronidase (60 U/mL), and DNase I (60 U/mL) solution. Cell suspension was passed through 70 µm cell strainer (Corning) and washed with FACS buffer. A negative control cell pull was left unstained. Cells were blocked with rat anti-mouse CD16/CD32 antibody mix (BD Biosciences), then stained with Ghost Dye Red 710 Viability Dye (Cell Signaling Technology). The staining mix containing CD11b-PE, F4/80-APC-Cy7, CD86-PerCP-Cy5.5, CD206-PE-Cy7 (BioLegend), CD38-FITC, and EGR2-APC (Thermo Fisher) antibodies was applied to the cells for 1 hour. Samples were washed and resuspended in FACS buffer. Flow cytometry analysis was performed with Aurora Spectral Analyzer (Cytek; Freemont, CA). UltraComp eBeads Compensation Beads (Thermo Fisher) bound with corresponding antibodies were used as single-stained controls. An unmixing matrix with autofluorescence extraction was calculated using SpectroFlo software (Cytek) and the samples were run using the live unmixing. Unmixed fcs files were analyzed using FlowJo v10.8 (BD Biosciences). Gating strategy shown in the **Supplementary Figure 2**.

Cultured spleen-derived monocytes, treated to induce M0, M[IFNγ+LPS] or M[IL4] phenotype, were lifted from the culture plates using Dispase (Thermo Fisher), washed with PBS, blocked with rat anti-mouse CD16/CD32 antibody mix (BD Biosciences), and stained with Ghost Dye Red 710 Viability Dye (Cell Signaling Technology). Cells were incubated in the antibody mix containing CD11b-PE, F4/80-APC-Cy7 (BioLegend), CD38-FITC, and EGR2-APC (Thermo Fisher) antibodies for 1 hour. Flow cytometry analysis was performed with Aurora Spectral Analyzer (Cytek) as described above.

### Mitochondrial function assessment

Macrophage mitochondrial bioenergetics was assessed using Seahorse XFe96 analyzer (Agilent Technologies; Santa Clara, CA) as described previously, with modifications (14). Live macrophage numbers were determined with Countess 3 FL Automated Cell Counter and 70000 cells/well were plated in Seahorse XFe96 cell culture microplates (Agilent). Attached cells were treated with either vehicle, 20 ng/mL IFNγ (PeproTech) and 10 ng/mL LPS (Sigma-Aldrich), or 20 ng/mL IL4 (PeproTech) for 24 hours. Before the assay, cells were washed, and media replaced with Seahorse XF RPMI medium supplemented with 10 mM glucose and 2 mM glutamine. Culture plate was placed in the CO_2_-free incubator for 1 hour before the test. Seahorse XFe96 Extracellular Flux Cartridge was hydrated with sterile water overnight in CO_2_-free incubator. Water was replaced with XF Calibrator 1 hour before the test. The oxygen consumption (OCR) and extracellular acidification rates (ECAR) were determined using Seahorse XFp Cell Mito Stress Test Kit (Agilent) consisting of 3 consecutive injections: 2 µM oligomycin (Oligo), 2 µM carbonyl cyanide-p-trifluoromethoxyphenylhydrazone (FCCP), and 1 µM rotenone with antimycin A (Rot/AA). Immediately after the test, cells were washed, lysed with M-PER Protein Extraction Reagent (Thermo Fisher), and protein concentration measured with Pierce BCA Protein Assay Kit (Thermo Fisher). The flux rates were normalized to µg protein per well. Mitochondria bioenergetics parameters were calculated as described previously (14). Metabolic profiles, mitochondrial and glycolytic contributions to ATP production rate were determined as described in (30).

### Plasma biochemical analyses and ELISA

Plasma cholesterol and triglycerides levels concentration were determined with AMS Liasys 330 Chemistry Analyzer (AMS Alliance; Weston, FL).

IL1β levels in conditional media were determined with Mouse Interleukin-1beta ELISA Kit (Thermo Fisher) following the manufacturer’s protocol.

### Real-time RT-PCR analysis

Total RNA was extracted from cultured macrophages using RNeasy Micro Kit (Qiagen; Germantown, MD), then cDNA was synthetized using iScript cDNA Synthesis Kit (Bio-Rad; Hercules, CA). Real-time PCR was performed with TaqMan GEN Expression Assays for *Nox4* (Mm00479246-m1) and *18S* (Hs99999901_s1), Universal PCR master mix (Thermo Fisher) and analyzed using 7500 Fast Real-Time PCR System (Thermo Fisher). Target RNA relative expression level was calculated by normalization to *18S* rRNA expression level.

### Statistical analysis

All analyses were done using Prism 9 (GraphPad Software; La Jolla, CA). All data were tested for normality with D’Agostino & Pearson test. Data were analyzed using unpaired t-test or one-way ANOVA with the Tukey multiple comparisons test, when appropriate. The results of OCR and ECAR measurements were analyzed using a two-way repeated measures ANOVA. All data presented as mean ± SEM. Differences were considered significant at p<0.05.

## Results

### *Nox4* deficiency reduces atherosclerosis burden and preserves plaque integrity in aged *Apoe* ^-/-^ mice

**Figure 1:**
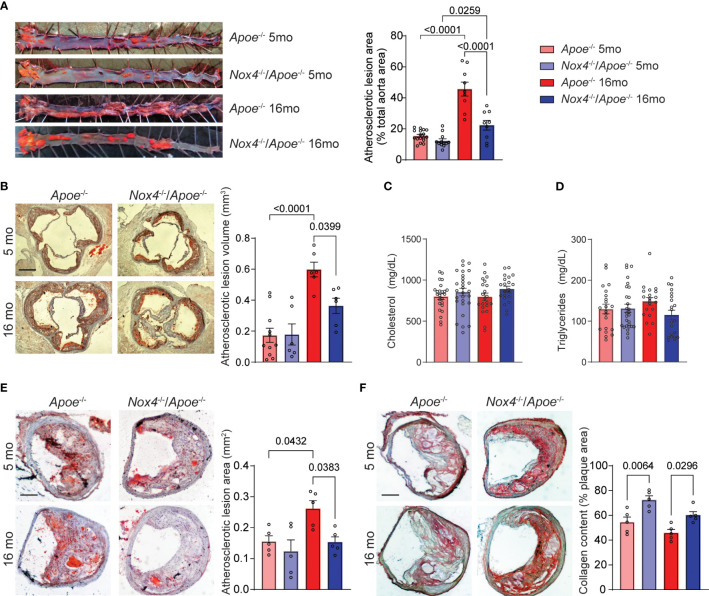
Aging-associated atherosclerosis burden is attenuated in *Nox4*-deficient *Apoe* ^-/-^ mice. **(A)** Representative images of oil red O-stained aortas and quantification of atherosclerotic lesion area in young (5-month-old) and aged (16-month-old) *Apoe* ^-/-^ and *Nox4* ^-/-^/*Apoe* ^-/-^ mice fed Western diet for 3 months (mean ± SEM, n=8). **(B)** Representative images of oil red O-stained aortic sinus sections and quantification of integrated atherosclerotic lesion volume (mean ± SEM, n=8). Plasma cholesterol **(C)** and triglyceride **(D)** levels in young and aged *Apoe* ^-/-^ and *Nox4* ^-/-^/*Apoe* ^-/-^ mice (mean ± SEM, n=20). **(E)** Representative images of oil red O-stained brachiocephalic artery sections and quantification of atherosclerotic lesion area (mean ± SEM, n=5). **(F)** Representative images of picrosirius red-stained brachiocephalic artery sections and quantification of atherosclerotic lesion collagen content (mean ± SEM, n=5).

The expression of NOX4 is increased in mouse and human atherosclerotic lesions with aging (14). To examine the impact of NOX4 on aging-associated atherosclerosis we initially determined atherosclerosis burden in four groups: young (5-month-old) and aged (16-month-old) *Apoe* ^-/-^ and *Nox4* ^-/-^/*Apoe* ^-/-^ mice. All the mice were fed Western diet for 3 months. The aortic atherosclerotic lesion area was comparable between young *Apoe* ^-/-^ and *Nox4* ^-/-^/*Apoe* ^-/-^ mice. Aged mice of both genotypes had significantly increased lesion area with *Apoe* ^-/-^ showing 195% increase and *Nox4* ^-/-^/*Apoe* ^-/-^ mice showing 82% increase. However, the lesion area was significantly lower in *Nox4* ^-/-^/*Apoe* ^-/-^ compared with *Apoe* ^-/-^ mice (p<0.0001; **Figure 1A**). Consistent with that, analysis of atherosclerotic lesion volume integrated from aortic sinus serial sections indicated similar lesion volume in young mice and significantly increased lesions in aged *Apoe* ^-/-^ but not in aged *Nox4* ^-/-^/*Apoe* ^-/-^ mice (p<0.0001 and p=0.1372, respectively; **Figure 1B**). Plasma cholesterol or triglyceride levels were not significantly different between young or aged *Apoe* ^-/-^ mice and their *Nox4* ^-/-^/*Apoe* ^-/-^ counterparts (**Figures 1C, D**). Mouse body weights increased with age but were not different between genotypes (**Supplementary Figure 3A**). There were no significant differences in plasma glucose or liver enzymes (**Supplementary Figures 3B–D**). Therefore, the observed increase in atherosclerosis cannot be attributed to metabolic changes, which underscores the role of NOX4 in the age-related progression of atherosclerotic lesions.

The progression of atherosclerotic lesions in the brachiocephalic (innominate) artery of older *Apoe* ^-/-^ mice is analogous to advanced human atherosclerotic disease (31). For that reason, we also evaluated atherosclerotic lesions in brachiocephalic/common carotid arteries of young and aged *Apoe* ^-/-^ and *Nox4* ^-/-^/*Apoe* ^-/-^ mice. We found no significant differences in the cross-sectional area of atherosclerotic lesions in the brachiocephalic arteries of young mice, consistent with our findings in aortic sinus lesions. However, as the *Apoe^-/-^* mice aged, the lesion area increased significantly, while the *Nox4^-/-^/Apoe^-/-^* mice showed no significant increase (p=0.0432 and p=0.8422, respectively; **Figure 1E**). Additionally, *Apoe^-/-^* mice showed a disintegrated fibrous cap and significantly lower plaque collagen content, as revealed by Picrosirius red staining (**Figure 1F**). In contrast, *Nox4*-deficient mice had preserved lesion fibrous cap and higher plaque collagen content compared with *Apoe* ^-/-^ mice. Although aging was associated with a slight decrease in collagen content, this change did not reach statistical significance (**Figure 1F**). These results suggest that aging is associated with a significant increase in atherosclerosis burden and the development of advanced plaque in *Apoe* ^-/-^ mice. In contrast, *Nox4* deletion reduced lesion size and preserved plaque integrity, indicating a critical role for NOX4 in aging-associated atherosclerosis.

### Aging is associated with increased NOX4-dependent ROS and oxidative DNA damage in atherosclerotic plaque

**Figure 2:**
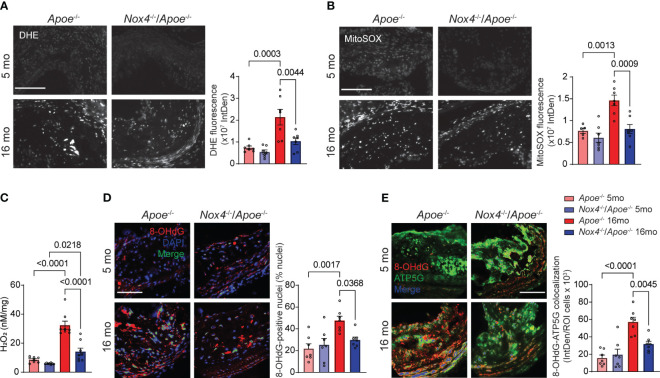
Atherosclerotic lesion mitochondrial ROS and oxidative DNA damage are reduced in aged *Nox4* ^-/-^/*Apoe* ^-/-^ as compared with aged *Apoe* ^-/-^ mice. **(A, B)** Representative fluorescence microscopy images and fluorescence quantification of brachiocephalic artery sections from young and aged *Apoe* ^-/-^ and *Nox4* ^-/-^/*Apoe* ^-/-^ mice stained with DHE **(A)** or MitoSOX **(B)**. Data are mean ± SEM, n=7. **(C)** Hydrogen peroxide levels in aortas were determined with AmplexRed assay. Data are H_2_O_2_ released per mg of dry tissue (mean ± SEM, n=8). **(D)** Representative fluorescence microscopy images and fluorescence colocalization quantification of 8-OHdG (red) with DAPI (blue). Data are percent of 8-OHdG-positive nuclei (green) (mean ± SEM, n=7). **(E)** Representative fluorescence microscopy images and fluorescence colocalization quantification of 8-OHdG (red) with ATP5G (green) in immunostained brachiocephalic artery sections. Data are fluorescence integrated density of mitochondria-localized 8-OHdG (blue) per lesion cell number (mean ± SEM, n=7).

Aging is linked to increased cellular and mitochondrial ROS, and oxidative DNA damage within atherosclerotic lesions of *Apoe* ^-/-^ mice (12, 14). Consistent with this, the ROS levels, determined by DHE fluorescence in brachiocephalic artery atherosclerotic lesions from *Apoe* ^-/-^ mice, significantly increased with age (p=0.0003; **Figure 2A**). Young *Nox4* ^-/-^/*Apoe* ^-/-^ exhibited ROS levels similar to those in *Apoe* ^-/-^ mice. However, in aged *Nox4* ^-/-^/*Apoe* ^-/-^ mice ROS levels were significantly lower than in their aged *Apoe* ^-/-^ counterparts (p=0.0044).

Similarly, mitochondrial ROS levels in brachiocephalic artery atherosclerotic lesions were not different between young *Apoe* ^-/-^ and *Nox4* ^-/-^/*Apoe* ^-/-^ mice when measured by MitoSOX fluorescence but increased significantly in aged *Apoe* ^-/-^ mice (p=0.0013; **Figure 2B**). Conversely, aged *Nox4* ^-/-^/*Apoe* ^-/-^ mice did not exhibit a significant change in mitochondrial ROS levels compared with young mice (p=0.5271; **Figure 2B**).

Given that NOX4 activity primarily generates hydrogen peroxide (32), we measured H_2_O_2_ release in freshly isolated aortas using the AmplexRed assay. Although no significant differences were observed in H_2_O_2_ levels in the aortas from young mice, hydrogen peroxide levels increased significantly in aged *Apoe* ^-/-^ and *Nox4* ^-/-^/*Apoe* ^-/-^ mice (p<0.0001 and p=0.0218, respectively; **Figure 2C**). Importantly, H_2_O_2_ levels in aged *Nox4* ^-/-^/*Apoe* ^-/-^ aortas were significantly lower than in aged *Apoe* ^-/-^ mice (p<0.0001). These results suggest that NOX4 is a key contributor to ROS production in aging-associated atherosclerosis.

The augmented oxidative stress in aging-associated atherosclerosis causes oxidative damage to both nuclear and mitochondrial DNA in vascular smooth muscle cells (VSMC) (12, 14). Assessment of 8-hydroxy-2'-deoxyguanosine (8-OHdG) levels in brachiocephalic artery atherosclerotic lesions showed a significant increase in 8-OHdG immunofluorescence colocalization with nuclear and mitochondrial DNA in aged *Apoe* ^-/-^ compared with young mice (**Figures 2D, E**). However, the age-associated increase in nuclear and mitochondrial 8-OHdG levels did not occur in aged *Nox4^-/-^/Apoe^-/-^* mice, supporting the notion that high NOX4 levels increase mitochondrial oxidative stress and cause DNA damage in aging atherosclerosis.

### NOX4 plays critical role in regulating vascular inflammation in aging-associated atherosclerosis

**Figure 3:**
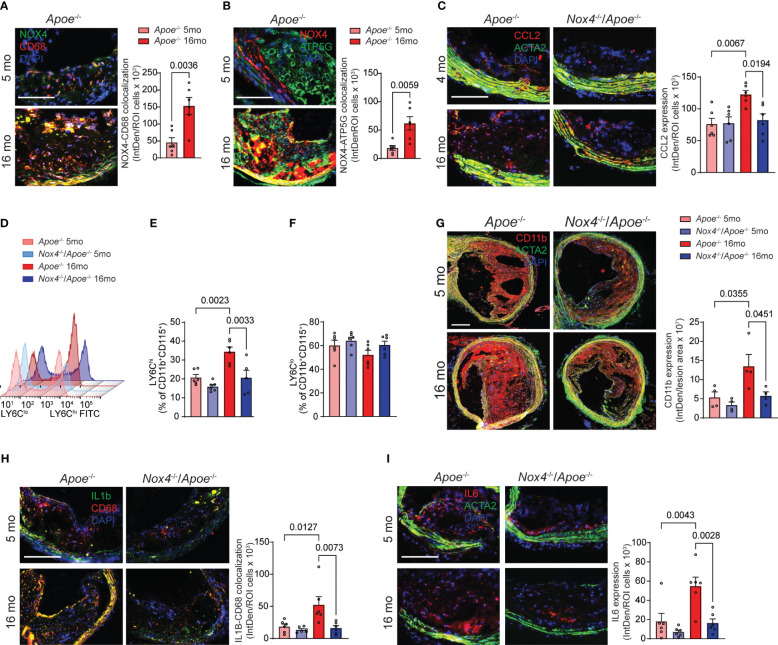
Increased NOX4 expression in aging as associated with vascular inflammation. **(A)** Representative fluorescence microscopy images and colocalization quantification of immunoreactive NOX4 (green) and CD68 (red) expression in brachiocephalic artery sections from young and aged *Apoe* ^-/-^ mice stained with DAPI (blue). Data are fluorescence colocalization integrated density per lesion cell number (mean ± SEM, n=6). **(B)** Representative fluorescence microscopy images and colocalization quantification of immunoreactive NOX4 (red) and ATP5G (green) in brachiocephalic artery sections stained with DAPI (blue). Data are fluorescence colocalization integrated density per lesion cell number (mean ± SEM, n=6). **(C)** Representative fluorescence microscopy images and quantification of immunoreactive CCL2 expression (red) in brachiocephalic artery sections stained for ACTA2 (green) and DAPI (blue). Data are fluorescence integrated density of CCL2 expression per lesion cell number (mean ± SEM, n=6). **(D)** Flow cytometry analysis of CD11b^+^CD115^+^Ly6C^+^ peripheral blood monocytes from young and aged *Apoe* ^-/-^ and *Nox4* ^-/-^/*Apoe* ^-/-^ mice. **(E)** Relative levels of Ly6C^hi^ peripheral blood CD11b^+^CD115^+^ monocytes. Data are mean ± SEM, n=6. **(F)** Relative levels of Ly6C^lo^ peripheral blood CD11b^+^CD115^+^ monocytes. Data are mean ± SEM, n=6. **(G)** Representative fluorescence microscopy images and quantification of immunoreactive CD11b expression (red) in brachiocephalic artery sections stained for ACTA2 (green) and DAPI (blue). Data are fluorescence integrated density of CD11b expression per lesion area (mean ± SEM, n=4). **(H)** Representative fluorescence microscopy images and colocalization quantification of immunoreactive IL1β (green) and CD68 (red) expression in brachiocephalic artery sections from young and aged *Apoe* ^-/-^ and *Nox4* ^-/-^/*Apoe* ^-/-^ mice stained with DAPI (blue). Data are fluorescence colocalization integrated density per lesion cell number (mean ± SEM, n=6). **(I)** Representative fluorescence microscopy images and quantification of immunoreactive IL6 expression (red) in brachiocephalic artery sections stained for ACTA2 (green) and DAPI (blue). Data are fluorescence integrated density of IL6 expression per lesion cell number (mean ± SEM, n=6).

Previous studies showed that inhibition of NOX4 or mitochondrial ROS in VSMC during aging atherosclerosis reduces vascular inflammation (14, 15). To further investigate this, we quantified aging-induced NOX4 expression in atherosclerotic plaque macrophages. Our analysis of brachiocephalic artery sections in aged *Apoe* ^-/-^ revealed a significant increase in expression of immunoreactive NOX4 colocalized with CD68^+^ cells compared with their young counterparts (a 230% increase; **Figure 3A**). This increase was most prominent in macrophages associated with the fibrous cap that is consistent with the vulnerable plaque phenotype. Furthermore, the relationship between NOX4 and mitochondrial oxidative stress in aging atherosclerosis was supported by substantially increased colocalization of immunoreactive NOX4 with mitochondrial marker ATP5G in brachiocephalic artery sections from aged *Apoe* ^-/-^ mice (a 3.3-fold increase over young *Apoe* ^-/-^ mice; **Figure 3B**).

CCL2-dependent monocytes recruitment to the atherosclerotic plaque is prominently involved in atherosclerosis progression and development of complications (33). Immunoreactive CCL2 expression was significantly higher in the atherosclerotic lesion sections from aged *Apoe* ^-/-^ compared with young *Apoe* ^-/-^ mice. The expression of CCL2 in aged *Apoe* ^-/-^ lesions was highest in fibrous cap-associated cells and medial SMC. In contrast, CCL2 expression in the plaques of young *Nox4*-deficient *Apoe* ^-/-^ mice was similar to that of young *Apoe* ^-/-^ mice and did not increase with age (**Figure 3C**). Flow cytometry analysis of peripheral blood cells showed that aged *Apoe^-/-^* mice had higher percentages of LY6C^hi^CD11b^+^CD115^+^ monocytes compared with young ones (p=0.0023; **Figures 3D, E**). Contrary to that, the percent of LY6C^hi^ monocytes was not different in aged compared with young *Nox4* ^-/-^/*Apoe* ^-/-^ mice but was significantly lower than that in aged *Apoe* ^-/-^ mice (p=0.0033; **Figures 3D, E**). Notably, the percentage of LY6C^lo^ monocytes was not different between young and aged mice (**Figure 3F**).

Consistent with that, immunofluorescent staining of brachiocephalic artery sections revealed that aged *Apoe^-/-^* mice had significantly more lesion infiltrating CD11b macrophages than young mice or aged *Nox4* ^-/-^/*Apoe* ^-/-^ mice (**Figure 3G**). These findings suggest that increased NOX4- and mitochondria-derived ROS in aging facilitate monocyte mobilization and recruitment in atherosclerotic lesions. Immunofluorescent staining further revealed significantly higher expression of immunoreactive IL1β and IL6 in atherosclerotic lesion in aged *Apoe* ^-/-^ compared with young mice or aged *Nox4* ^-/-^/*Apoe* ^-/-^ mice (**Figures 3H, I**). Notably, higher expression of IL1β in aged *Apoe* ^-/-^ mice plaques was localized with the cells proximal to the fibrous cap and medial cells, whereas IL6 expression was higher in the plaque core cells and medial SMC. These observations indicate that increased mitochondrial oxidative stress in aging-associated atherosclerosis mediates monocyte mobilization/recruitment and intraplaque inflammation. Conversely, the deletion of *Nox4* reduces plaque macrophage infiltration and inflammation.

### Aging induces proinflammatory phenotype whereas *Nox4* deficiency favors pro-resolving phenotype in atherosclerotic lesion macrophages in *Apoe* ^-/-^ mice

**Figure 4:**
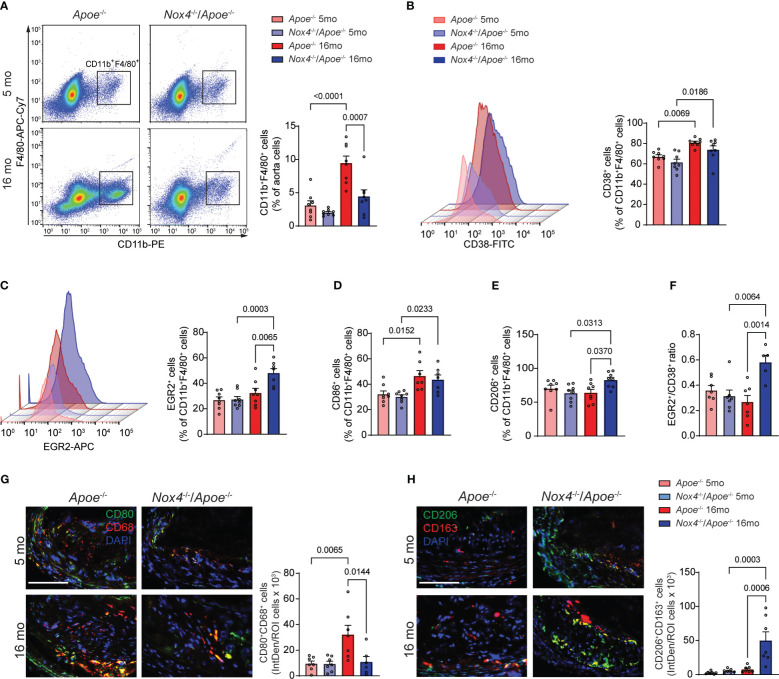
*Nox4* deficiency induces pro-resolving phenotype in atherosclerotic lesion macrophages in aged *Apoe* ^-/-^ mice. **(A)** Flow cytometry analysis and quantification of atherosclerotic lesion single cell suspension from young and aged *Apoe* ^-/-^ and *Nox4* ^-/-^/*Apoe* ^-/-^ mice for CD11b^+^F4/80^+^ macrophages. Data are macrophage fraction of aorta cells (mean ± SEM, n=6). **(B, C)** Flow cytometry analysis and quantification of CD38^+^ **(B)** and EGR2^+^ **(C)** cell fraction of CD11b^+^F4/80^+^ aorta macrophages (mean ± SEM, n=6). **(D, E)** Flow cytometry quantification of CD86^+^ **(D)** and CD206^+^ **(E)** cell fractions of CD11b^+^F4/80^+^ aorta macrophages (mean ± SEM, n=6). **(F)** Ratio of EGR2^+^ to CD38^+^ cells (mean ± SEM, n=6). **(G)** Representative fluorescence microscopy images and quantification of CD80^+^ (green) CD68^+^ (red) macrophages in brachiocephalic artery sections from young and aged *Apoe* ^-/-^ and *Nox4* ^-/-^/*Apoe* ^-/-^ mice. Data are fluorescence colocalization integrated density per lesion cell number (mean ± SEM, n=6). **(H)** Representative fluorescence microscopy images and quantification of CD206^+^ (green) CD163^+^ (red) macrophages in brachiocephalic artery sections counterstained with DAPI (blue) from young and aged *Apoe* ^-/-^ and *Nox4* ^-/-^/*Apoe* ^-/-^ mice. Data are fluorescence colocalization integrated density per lesion cell number (mean ± SEM, n=6).

To better understand the inflammatory phenotype of the atherosclerotic lesion macrophages, we conducted flow cytometry analysis of cells obtained from aortas of young and aged *Apoe* ^-/-^ and *Nox4* ^-/-^/*Apoe* ^-/-^ mice fed Western diet (24). First, we determined the percentage of CD11b^+^F4/80^+^ macrophages in aortic cell suspensions. Consistent with the atherosclerotic lesion size, the proportion of macrophages was similar in young *Apoe* ^-/-^ and *Nox4* ^-/-^/*Apoe* ^-/-^ mice (**Figure 4A**). However, the macrophage fraction of the aortic cells increased significantly with age and was 183% higher in aged *Apoe* ^-/-^ than in *Nox4* ^-/-^/*Apoe* ^-/-^ mice (p=0.0007; **Figure 4A**).

In an effort to distinguish between classically (M[IFNγ+LPS]) and alternatively activated (M[IL4]) macrophages, we used specific markers for flow cytometry analysis - CD38 and EGR2, respectively (34). The proportion of CD38^+^ among CD11b^+^F4/80^+^ aortic cells was not different between genotypes and significantly increased with age in both *Apoe* ^-/-^ and *Nox4* ^-/-^/*Apoe* ^-/-^ mice (**Figure 4B**). Conversely, the proportion of EGR2^+^ M[IL4] aorta macrophages was not different in young mice and significantly increased with age in *Nox4* ^-/-^/*Apoe* ^-/-^ but not in *Apoe* ^-/-^ mice (**Figure 4C**). This pattern was mirrored in the analysis of the CD86^+^ M[IFNγ+LPS] macrophages that demonstrated significant aging-associated increases in both genotypes, whereas the CD206^+^ M[IL4] aorta macrophage proportion significantly increased with age only in *Nox4* ^-/-^/*Apoe* ^-/-^ but not in *Apoe* ^-/-^ mice (**Figures 4D, E**). Moreover, the EGR2^+^/CD86^+^ macrophage ratio in atherosclerotic aortas was not different in young mice and increased with age solely in *Nox4* ^-/-^/*Apoe* ^-/-^ mice (**Figure 4F**). The relative increase of classically activated proinflammatory macrophages fraction was evident in atherosclerotic lesions of both genotypes. However, because the number of CD11b^+^F4/80^+^ macrophages in atherosclerotic aortas was significantly decreased in aged *Nox4* ^-/-^/*Apoe* ^-/-^ mice, the proportion of CD38^+^CD86^+^ macrophages was also relatively lower compared with that in aged *Apoe* ^-/-^ mice.

To corroborate these findings, we performed immunofluorescent staining of the brachiocephalic artery sections from young and aged *Apoe* ^-/-^ and *Nox4* ^-/-^/*Apoe* ^-/-^ mice to determine the relative abondance of M[IFNγ+LPS] or M[IL4] macrophages within the atherosclerotic plaques. Immunofluorescence analysis revealed that young mice in both groups had a similar number of CD80^+^CD68^+^ (M[IFNγ+LPS]) cells (**Figure 4G**). However, the atherosclerotic plaques of aged *Apoe* ^-/-^ mice had a significantly higher proportion of CD80^+^CD68^+^ plaque cells compared with young mice or aged *Nox4* ^-/-^/*Apoe* ^-/-^ mice (p=0.0144). Furthermore, immunofluorescence staining for CD206 and CD163 displayed no differences in atherosclerotic plaques from young mice. Nonetheless, there was a substantially higher proportion of CD206^+^CD163^+^ cells in the brachiocephalic artery plaques from aged *Nox4* ^-/-^/*Apoe* ^-/-^ compared with aged *Apoe* ^-/-^ mice (p=0.0006; **Figure 4H**). Taken together, these results suggest that the number of atherosclerotic lesion macrophages increases in aged *Apoe* ^-/-^ mice, predominantly comprising of classically activated pro-inflammatory macrophages. In contrast, *Nox4* deficiency in aged *Apoe* ^-/-^ mice results in reduced macrophage infiltration in the plaque and increased proportion of pro-resolving macrophages. This combination leads to attenuated atherosclerotic lesion size and lower intraplaque inflammation.

### NOX4 mediates mitochondrial dysfunction and glycolytic switch in aged *Apoe* ^-/-^ mice monocyte-derived macrophages

**Figure 5:**
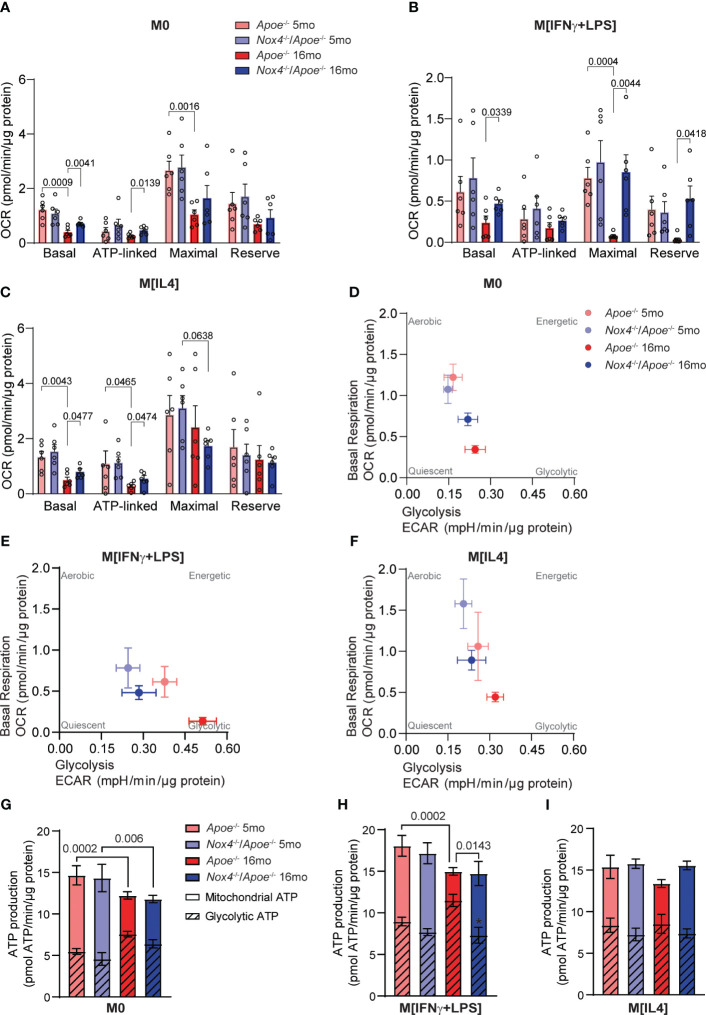
Mitochondrial function and metabolic profiling of macrophages from young and aged *Apoe* ^-/-^ and *Nox4* ^-/-^/*Apoe* ^-/-^ mice. **(A-C)** Oxygen consumption rate (OCR) measurements and mitochondria bioenergetic parameters were determined in control M0 **(A)**, M[IFNγ+LPS] **(B)**, and M[IL4] **(C)** cultured macrophages isolated from young and aged *Apoe* ^-/-^ and *Nox4* ^-/-^/*Apoe* ^-/-^ mice using Agilent Seahorse XF96 analyzer (mean ± SEM, n=6). **(D, E)** Metabolic profiling showing basal respiration and glycolysis relations in control M0 **(D)**, M[IFNγ+LPS] **(E)**, and M[IL4] **(F)** cultured macrophages isolated from young and aged *Apoe* ^-/-^ and *Nox4* ^-/-^/*Apoe* ^-/-^ mice (mean ± SEM, n=6). **(G-I)** Mitochondrial and glycolytic contribution to ATP production in control M0 **(G)**, M[IFNγ+LPS] **(H)**, and M[IL4] **(I)** cultured macrophages isolated from young and aged *Apoe* ^-/-^ and *Nox4* ^-/-^/*Apoe* ^-/-^ mice (mean ± SEM, n=6).

Mitochondrial dysfunction and metabolic switch are implicated in pro-inflammatory priming of peripheral monocytes and subsequent changes in macrophage phenotypes within atherosclerotic plaques (8). The spleen is a source of monocytes that infiltrate atherosclerotic lesions (35), so we used spleen-derived monocytes from young and aged *Apoe* ^-/-^ and *Nox4* ^-/-^/*Apoe* ^-/-^ mice to assess macrophage mitochondrial bioenergetics. We measured the oxygen consumption rate (OCR) and extracellular acidification rate (ECAR) to assess mitochondrial respiration and cellular metabolic rate in M0, M[IFNγ+LPS] or M[IL4] cells. Our results showed that mitochondrial respiration in control M0 and M[IL4] monocyte-derived macrophages from young mice was similar (**Supplementary Figures 4A, B, E, F**). However, with aging, there was a significant decline in OCR in both M0 and M[IL4] macrophages, and an increase in ECAR in M0 macrophages. Both basal and ATP-linked respiration were significantly lower in aged *Apoe* ^-/-^ M0 and M[IL4] as compared with the cells from young mice or aged *Nox4* ^-/-^/*Apoe* ^-/-^ mice (**Figures 5A, C**). Furthermore, mitochondrial respiration was lower in M[IFNγ+LPS] as compared with M0 or M[IL4] macrophages (**Supplementary Figure 4C**). In M[IFNγ+LPS] monocyte-derived macrophages, OCR and ECAR were similar in young *Apoe* ^-/-^ and *Nox4* ^-/-^/*Apoe* ^-/-^ mice. However, aging significantly downregulated OCR and upregulated ECAR in *Apoe* ^-/-^ but not in *Nox4* ^-/-^/*Apoe* ^-/-^ monocyte-derived macrophages (**Supplementary Figures 4C, D**). Basal, maximal, and reserve respiration rates in aged *Apoe* ^-/-^ M[IFNγ+LPS] were significantly lower than that in aged *Nox4* ^-/-^/*Apoe* ^-/-^ macrophages (**Figure 5B**).

The ratio of OCR/ECAR greater than 4 indicates the preference for oxidative phosphorylation over glycolysis for energy production. In macrophages derived from aged *Apoe* ^-/-^ monocytes the OCR/ECAR ratio was below 4, which shows a preference for glycolysis, in contrast with cells from their young counterparts or *Nox4*-deficient mice (**Supplementary Figure 4G**). In line with that, metabolic profiling of M0, M[IFNγ+LPS] or M[IL4] macrophages derived from aged *Apoe* ^-/-^ monocytes exhibited a similar pattern characterized by lower OCR, higher ECAR, and a greater reliance on glycolysis (**Figures 5D–F**).

Our calculations of mitochondrial oxidative phosphorylation and glycolysis contribution to the ATP production also showed that aging significantly increased glycolytic ATP and reciprocally decreased mitochondrial ATP production in M0 and M[IFNγ+LPS] macrophages derived from aged *Apoe* ^-/-^ monocytes (**Figures 5C–H**). Taken together, these data suggest that higher NOX4 activity and mitochondrial oxidative stress in aging were synergistically associated with M[IFNγ+LPS] pro-inflammatory macrophage phenotype, reduced mitochondrial respiration and increased glycolysis. On the other hand, the absence of *Nox4* in aging was associated with M[IL4] pro-resolving macrophage phenotype and preserved mitochondrial function.

### *Nox4* deficiency in aging is associated with reduced mitochondrial oxidative stress and pro-resolving phenotype in monocyte-derived macrophages

In our previous studies we found that aging is associated with a significant increase in NOX4-dependent cellular and mitochondrial ROS in atherosclerotic lesions (13–15). *In situ* ROS measurements suggested that macrophages are significant contributors to the intraplaque oxidative stress and inflammation. To understand the role of oxidative stress in macrophage phenotype changes, we measured ROS levels in spleen-derived M0, M[IFNγ+LPS] or M[IL4] cells from young and aged *Apoe* ^-/-^ and *Nox4* ^-/-^/*Apoe* ^-/-^ mice.

**Figure 6:**
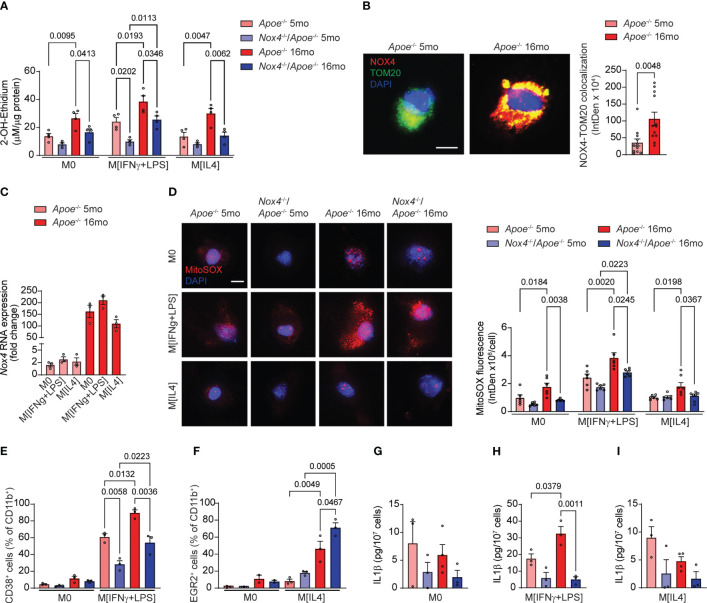
Macrophages from aged *Nox4*-deficient *Apoe* ^-/-^ mice acquire pro-resolving phenotype associated with lower mitochondrial ROS and decreased inflammasome activation. **(A)** Superoxide generation in control M0, M[IFNγ+LPS]-treated, and M[IL4]-treated cultured macrophages isolated from young and aged *Apoe* ^-/-^ and *Nox4* ^-/-^/*Apoe* ^-/-^ mice was measured with 2-OH-ethidium HPLC analysis (mean ± SEM, n=4). **(B)** Representative fluorescence confocal microscopy images of macrophages isolated from young and aged *Apoe* ^-/-^ mice and stained for immunoreactive NOX4 (red) and TOM20 (green) and counterstained with DAPI (blue). Quantification of mitochondrial NOX4 and TOM20 colocalization presented as fluorescence integrated density per cell (mean ± SEM, n=6). **(C)** Real-time RT-PCR analysis of Nox4 mRNA levels in control M0, M[IFNγ+LPS]-treated, and M[IL4]-treated cultured macrophages isolated from young and aged *Apoe* ^-/-^ mice. Data are expression fold change relative to control M0 macrophage levels (mean ± SEM, n=3). **(D)** Representative fluorescence confocal microscopy images and quantification of MitoSOX fluorescence in control M0, M[IFNγ+LPS]-treated, and M[IL4]-treated cultured macrophages isolated from young and aged *Apoe* ^-/-^ and *Nox4* ^-/-^/*Apoe* ^-/-^ mice. Data are MitoSOX fluorescence integrated density per cell (mean ± SEM, n=4). **(E, F)** Flow cytometry analysis of CD38^+^ **(E)** or EGR2^+^ **(F)** cell fractions of CD11b^+^ control M0 and M[IFNγ+LPS] **(E)** or control M0 and M[IL4] **(F)** cultured macrophages isolated from young and aged *Apoe* ^-/-^ and *Nox4* ^-/-^/*Apoe* ^-/-^ mice (mean ± SEM, n=6). **(G-I)** Concentration of IL1β in conditional media from control M0 **(G)**, M[IFNγ+LPS]-treated **(H)**, and M[IL4]-treated **(I)** cultured macrophages isolated from young and aged *Apoe* ^-/-^ and *Nox4* ^-/-^/*Apoe* ^-/-^ mice determined with ELISA (mean ± SEM, n=3–4).

We first measured superoxide (O_2_^•−^) generation in macrophages by HPLC analysis of 2-hydroxyethidium. The O_2_^•−^-dependent levels of 2-OH-ethidium were not different in M0 or M[IL4] macrophages from young mice of both genotypes but were significantly higher in *Apoe* ^-/-^ than in *Nox4* ^-/-^/*Apoe* ^-/-^ M[IFNγ+LPS] macrophages (**Figure 6A**). The levels of O_2_^•−^ in *Apoe^-/–^* derived macrophages increased significantly with age. Similarly, O_2_^•−^ levels associated with aging were significantly higher in Nox4-deficient M[IFNγ+LPS] macrophages. However, O_2_^•−^ levels were lower in Nox4-deficient M0 and M[IL4] macrophages as compared with the aged *Apoe^-/–^* derived macrophages (**Figure 6A**).

Immunofluorescence analysis of *Apoe* ^-/-^ macrophages indicated that mitochondrial NOX4 expression levels (NOX4 colocalized with TOM20) were significantly higher in aged than in young cells (**Figure 6B**). Congruently, *Nox4* mRNA expression increased with aging in *Apoe* ^-/–^derived monocytes by 80-fold on average (**Figure 6C**). However, *Nox4* expression levels were not directly affected by macrophage polarization and did not differ between M0, M[IFNγ+LPS] or M[IL4] macrophages from either young or aged mice.

Analysis of MitoSOX fluorescence indicated no significant differences in mitochondrial ROS levels in macrophages from young mice (**Figure 6D**). M0, M[IFNγ+LPS] and M[IL4] macrophages from aged *Apoe* ^-/-^ mice had significantly higher mitochondrial ROS levels than cells from the young mice. In contrast, mitochondrial ROS increased with age only in M[IFNγ+LPS] *Nox4* ^-/-^/*Apoe* ^-/-^ macrophages but remained significantly lower than in aged *Apoe* ^-/-^ (**Figure 6D**). Similarly, M[IL4] aged *Nox4* ^-/-^/*Apoe* ^-/-^ macrophages had lower mitochondrial ROS levels than in aged M[IFNγ+LPS] *Nox4* ^-/-^/*Apoe* ^-/-^ macrophages. (**Figure 6D**). However, M[IL4] macrophages showed lower mitochondrial ROS levels than M[IFNγ+LPS] macrophages in both young and aged mice.

We conducted further tests to determine whether macrophage polarization is affected by NOX4-derived mitochondrial ROS. Flow cytometry analysis of spleen-derived monocytes from young and aged *Apoe* ^-/-^ and *Nox4* ^-/-^/*Apoe* ^-/-^ mice did not show differences in the percentage of CD38^+^ or EGR2^+^ cells in CD11b^+^ M0 macrophages (**Figures 6E, F**). The percentage of CD38^+^CD11b^+^ cells increased with age in mice of both genotypes but was significantly more so in *Apoe* ^-/-^ then in *Nox4* ^-/-^/*Apoe* ^-/-^ mice (**Figure 6E**). In contrast, the percentage of EGR2^+^CD11b^+^ M[IL4] was comparable between the genotypes in macrophages from young mice, but significantly increased in macrophages from aged mice. The increase was significantly higher in aged *Nox4* ^-/-^/*Apoe* ^-/-^ compared with *Apoe* ^-/-^ counterparts (**Figure 6F**).

Because increased mitochondrial ROS and dysfunction in aging leads to inflammasome activation (36) and is associated with pro-inflammatory macrophage phenotype (37), we assessed inflammasome activity by measuring IL1β levels in conditional media from M0, M[IFNγ+LPS], and M[IL4] monocyte-derived macrophages. There were no significant differences in IL1β levels in M0 or M[IL4] macrophages derived from monocytes of young or aged mice of both genotypes (**Figures 6G, I**). However, M[IFNγ+LPS] macrophages from aged *Apoe* ^-/-^ mice had significantly higher secreted IL1β levels than in macrophages from young *Apoe* ^-/-^ or aged *Nox4* ^-/-^/*Apoe* ^-/-^ mice (**Figure 6H**).

Our results indicate that increased NOX4 activity in aging induces mitochondrial oxidative stress and dysfunction, thereby inducing glycolytic metabolic switch, inflammasome activation and priming monocytes to acquire pro-inflammatory phenotype. However, if NOX4 activity is reduced during aging, mitochondrial function is preserved, inflammasome activation is reduced, and a higher percentage of monocytes acquire a pro-resolving phenotype.

### Inhibiting NOX4 activity in aging improves mitochondrial function and induces pro-resolving phenotype in *Apoe* ^-/-^ macrophages

**Figure 7:**
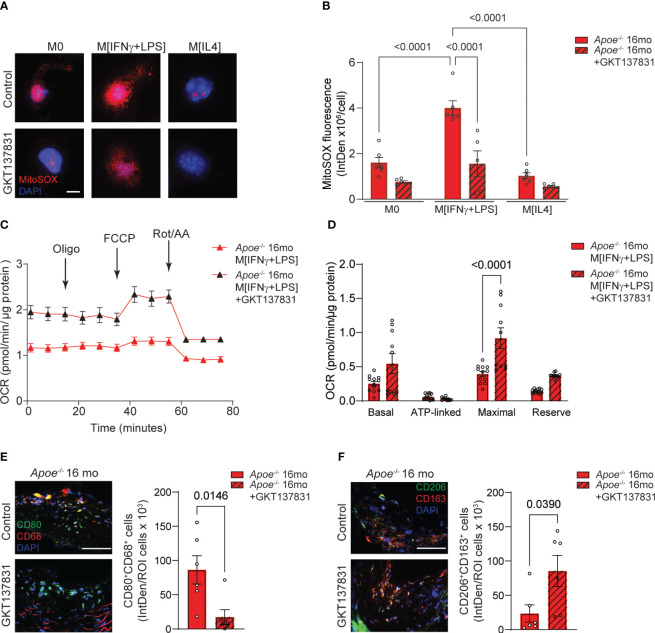
Inhibition of NOX4 improves mitochondrial function inducing pro-resolving phenotype in atherosclerotic lesion macrophages in aged *Apoe* ^-/-^ mice. **(A)** Representative fluorescence confocal microscopy images of MitoSOX (red) and DAPI (blue) stained control M0, M[IFNγ+LPS]- or M[IL4]-treated macrophages from aged *Apoe* ^-/-^ mice pre-treated with vehicle or 10 µM GKT137831. **(B)** Quantification of MitoSOX fluorescence in cultured control M0, M[IFNγ+LPS]- or M[IL4]-treated macrophages pre-treated with vehicle or GKT137831. Data are fluorescence integrated density per cell (mean ± SEM, n=6). **(C)** Mitochondrial oxygen consumption rate (OCR) was measured in aged *Apoe* ^-/-^ M[IFNγ+LPS]-treated macrophages pre-treated with vehicle or GKT137831 using Agilent Seahorse XF96 analyzer (mean ± SEM, n=12). **(D)** Mitochondrial bioenergetic parameters were derived from OCR measurements in aged *Apoe* ^-/-^ M[IFNγ+LPS]-treated macrophages pre-treated with vehicle or GKT137831 (mean ± SEM, n=12). **(E, F)** Representative fluorescence microscopy images and quantification of CD80^+^ (green) CD68^+^ (red) **(E)** and CD206^+^ (green) CD163^+^ (red) **(F)** macrophages in brachiocephalic artery sections counterstained with DAPI (blue) from aged *Apoe* ^-/-^ mice treated with GKT137831. Data are fluorescence colocalization integrated density per lesion cell number (mean ± SEM, n=6).

We have previously demonstrated that using a pharmacological inhibitor that targets NOX4/NOX1 activity in aged *Apoe* ^-/-^ mice led to a decrease in atherosclerotic lesion size, VSMC mitochondrial ROS levels, and inflammation (14, 15). These studies underscored the role of VSMC NOX4 in the development of cardiovascular pathology associated with aging. To investigate the effects of NOX4 inhibitor on macrophage mitochondrial function and metabolic phenotype in atherosclerosis, we first measured the levels of mitochondrial ROS in macrophages derived from aged *Apoe* ^-/-^ mice (**Figure 7A**). The MitoSOX fluorescence was significantly higher in M[IFNγ+LPS] compared with M0 or M[IL4] macrophages. Treatment with GKT137831 significantly abrogated MitoSOX fluorescence in M[IFNγ+LPS] macrophages from aged *Apoe* ^-/-^ mice (p<0.0001; **Figure 7B**). Consistent with increased ROS levels, mitochondrial OCR was substantially reduced in M[IFNγ+LPS] macrophages from aged *Apoe* ^-/-^ mice as compared with M0 or M[IL4] (**Figure 7C**; **Supplementary Figures 5A, C**). However, GKT137831 treatment significantly improved mitochondrial function by increasing both OCR and maximal respiration (**Figures 7C, D**). Similarly, the GKT137831 significantly enhanced reserved respiration in M0 or M[IL4] and maximal respiration in M0 macrophages from aged *Apoe* ^-/-^ mice (**Supplementary Figures 5B, D**). Consistent with our observations in aged *Nox4* ^-/-^/*Apoe* ^-/-^ mice, the treatment with NOX4 inhibitor in aged *Apoe* ^-/-^ mice significantly reduced the number of CD68^+^CD80^+^ (**Figure 7E**) and increased the number of CD206^+^CD163^+^ (**Figure 7F**) macrophages observed in atherosclerotic lesion sections.

Taken together, these data suggest that similar to genetic deletion of Nox4, the inhibition of NOX4 activity reduces mitochondrial ROS levels and preserves function in M[IFNγ+LPS] macrophages, thereby limiting the expansion of pro-inflammatory macrophage population in the atherosclerotic lesion. Conversely, NOX4 inhibition significantly increases the proportion of pro-resolving macrophages, a trend that aligns with the reduction in vascular inflammation and atherosclerotic lesion area in aged *Apoe* ^-/-^ mice.

## Discussion

In the present study, we assessed the relation between NOX4-dependent mitochondrial oxidative stress and macrophage metabolic switch-induced pro-inflammatory phenotype in the progression of atherosclerotic lesions in aging. Our results show that: 1) increased NOX4 activity in macrophages during aging results in the pro-inflammatory phenotype by inducing mitochondrial oxidative stress and dysfunction and switching the metabolism to glycolysis; 2) aged *Apoe* ^-/-^ mice exhibit increased proportions of classically activated pro-inflammatory macrophages in atherosclerotic plaques, whereas *Nox4*-deficient *Apoe* ^-/-^ mice show reduced plaque macrophage infiltration and a higher proportion of pro-resolving macrophages; 3) increased NOX4 and mitochondrial ROS levels facilitate increased intraplaque inflammation, monocyte recruitment, and lesion expansion in aging-associated atherosclerosis; and 4) *Nox4*-deficient *Apoe* ^-/-^ mice show significantly reduced atherosclerotic burden compared with aged *Apoe* ^-/-^ mice, demonstrating that deficiency *of Nox4* slows down the progression of atherosclerosis in aging. These results were corroborated by pharmacological inhibition of NOX4 activity in aged *Apoe* ^-/-^ mice that significantly reduced mitochondrial ROS levels, improved mitochondrial function, and promoted pro-resolving phenotype in plaque macrophages, reducing inflammation, lesion size and complexity.

The expression and activity of NOX4 is increased in human and mouse atherosclerotic lesions and SMC with age and is independent of diet (14, 15, 19). Our results confirm these observations and show higher NOX4 levels in atherosclerotic lesions, particularly in lesional macrophages of aged *Apoe* ^-/-^ mice. Transcriptional activation of *Nox4* in aging may result from increased activity of inflammation-associated transcription factors such as NF-κB (38) or STAT1/3 (39). Alternatively, epigenetic regulation of *Nox4* through increased acetylation of H4K16ac or methylation of H4K20me3, both of which are increased in aging, was also reported (40). Similar upregulation of NOX4 is possible in monocytes/macrophages, which we observed, given that monocytes are more susceptible to epigenetic chromatin modifications in aging than other immune cells (41).

Increased ROS generation from elevated NOX1 expression and activation has been observed during the initial stages of atherogenesis in young hypercholesterolemic mice (24, 42, 43). Similarly, NOX2 expression in monocytes/macrophages and endothelial cells has been linked to early atherogenesis in young *Apoe* ^-/-^ mice (44, 45). On the other hand, expression of NOX4, but not NOX1 or NOX2, increased in the arterial VSMC of aged *Apoe* ^-/-^ mice, with higher ROS levels and mitochondrial DNA damage correlating with NOX4 expression and activity in advanced atherosclerotic lesions (14–16). Increased mitochondrial DNA damage in both vessel walls and circulating cells is causative in atherosclerosis (46). Our current data align with these findings, showing that deleting *Nox4* in young *Apoe* ^-/-^ mice did not significantly alter atherosclerotic lesion size or ROS levels. However, lifelong *Nox4* deficiency significantly attenuated plaque mitochondrial ROS levels, oxidative DNA damage, and lesion expansion during aging. On the other hand, young hypercholesterolemic mice with Nox4 deletion exhibited an increase in atherosclerosis (47, 48), while endothelial-specific NOX4 overexpression protected against atherosclerosis (49). These findings indicate that the effects of NOX4 on atherosclerosis are age- and tissue-specific.

NOX4-derived ROS stimulate collagen synthesis and the expression of activated myofibroblast markers that are induced by TGFβ1 (50). We have previously reported that TGFβ1 induces NOX4 expression in atherosclerotic lesions in aging (15). TGFβ1 can control the transcription of *Nox4* in VSMC by activating TAK1 (MAP3K7) or alternative pathways, such as RELA/NF-κB or cJUN/AP1 (15). TGFβ1 also enhances the expression and activity of NOX4 in human aortic SMC (51), suggesting a feedback loop. This interplay is evident in aged human atherosclerotic lesions, where TGFβ1 expression is correlated with NOX4 levels (15). Furthermore, aging is characterized by an increase in NOX4-dependent mitochondrial ROS and compromised mitochondrial function in aortic wall cells, leading to augmented collagen content and aortic stiffness (14). Excessive mitochondrial ROS, induced by NOX4 activity, plays a role in the progression and instability of atherosclerotic plaques by enhancing MMP2 activity. This leads to the degradation of the extracellular matrix (ECM) and collagen, ultimately contributing to fibrous cap rupture in aged mice (12, 52).

NOX4-derived reactive oxygen species (ROS) are crucial in maintaining VSMC differentiated phenotype (53). On the other hand, the deletion of NOX4 results in the dedifferentiation and proliferation of VSMCs in diabetic *Apoe^-/-^* mice (54). Xu et al. have shown that advanced atherosclerotic lesions exhibit increased Nox4 levels, resulting in SMC apoptosis and an unstable plaque phenotype (16). Our results showed that aging alone did not induce any features of plaque rupture or intraplaque hemorrhage in *Apoe^-/-^* mice. However, we observed that atherosclerotic lesions in aged *Nox4^-/-^/Apoe^-/-^* mice had preserved fibromuscular caps with many synthetic VSMCs and increased collagen content compared with their *Apoe^-/-^* counterparts.

Increased NOX4 expression in aged *Apoe^-/-^* mice is associated with elevated IL1β expression in the fibrous cap and medial cells of atherosclerotic plaques. IL1β promotes the expression of many inflammatory mediators, including IL6 and matrix metalloproteinases, that can cause plaque rupture (55). In experimental atherosclerosis, neutralizing IL1β promotes monocytes to switch to a less inflammatory state, reducing plaque size (56). A large clinical study, CANTOS, demonstrated the critical role of IL1β in promoting atherosclerosis and the effectiveness of anti-inflammatory therapy in treating it (4). Increased IL6 expression in the plaque core cells and medial SMC of aged *Apoe^-/-^* mice is consistent with prior findings that NOX4 and mitochondrial ROS promote IL-6 expression and activity (25, 57). IL6 induces acute phase response, increasing reactants like CRP, fibrinogen, and plasminogen activator inhibitors, closely linked to atherothrombosis (55).

Macrophages can polarize into pro-inflammatory and pro-resolving macrophages in response to environmental stimuli by mitochondrial metabolism and physiology changes. Aged Apoe^-/-^ mice with increased NOX4 expression had a higher percentage of classically activated pro-inflammatory macrophages (CD38^+^CD80^+^), while Nox4^-/-^/Apoe^-/-^ mice had a higher proportion of alternatively activated pro-resolving macrophages (EGR2^+^/CD163^+^CD206^+^) in the lesions. Additionally, macrophages from aged *Apoe^-/-^* mice had higher total and mitochondrial superoxide levels when splenic monocyte polarization was induced with IFNγ+LPS or IL4, compared to Nox4^-/-^/Apoe^-/-^ mice. However, the expression of NOX4 was not affected by macrophage polarization as NOX4 levels are already high during aging. The increased expression of inflammasome activation markers in aged *Apoe^-/-^* mouse atherosclerotic lesions compared with *Nox4^-/-^/Apoe^-/-^* mice is consistent with increased mitochondrial oxidative stress, increased number of CD38^+^CD80^+^ inflammatory macrophages, and impaired mitochondrial respiration (34).

Macrophage polarization is a dynamic process that is regulated by metabolism. Alternatively activated M[IL4] macrophages exhibit enhanced mitochondrial oxidative metabolism compared to inflammatory macrophages (58). Apart from glucose, M[IL4] macrophages also metabolize fatty acids. M[IL4] macrophages express arginase which breaks down arginine into ornithine and urea (59). Ornithine is pivotal in polyamine and proline synthesis, which are crucial for cell proliferation and tissue repair. Inhibition of fatty acid oxidation (FAO) and/or OXPHOS decreases arginase activity in macrophages and inhibits M[IL4] polarization (60, 61). The altered metabolic status that occurs with M[IL4] differentiation is underpinned by the activation of FAO and mitochondrial biogenesis-related pathways induced by peroxisome proliferator-activated receptor (PPARγ) and PPARγ coactivator 1β (PGC1β) (61). Reduced mitochondrial oxidative stress and dysfunction in aged *Nox4* ^-/-^/*Apoe* ^-/-^ mice potentially promote the polarization of macrophages to M[IL4] phenotype and proliferation.

Pro-inflammatory M[IFNγ+LPS] macrophages are predominantly glycolytic, whereas pro-resolving M[IL4] macrophages are highly oxidative, supporting the role of metabolism in macrophage polarization (62). Increased flux of glycolytic metabolites enters the pentose phosphate pathway, producing NADPH, which activates NADPH oxidase and increases ROS generation. The metabolic shift may not initiate inflammation but primes macrophages for a greater inflammatory response, as seen in hyperlipidemic conditions during aging. GKT137831/Setanaxib treatment, which reduced the atherosclerotic burden in aged *Apoe^-/-^* mice (14), increased maximal respiration in splenic M[IFNγ+LPS] macrophages while decreasing mitochondrial oxidative stress. In addition, GKT137831/Setanaxib significantly decreased the number of CD38^+^CD80^+^ macrophages while increasing the CD163^+^CD206^+^ macrophage population in aged *Apoe^-/-^* mice.

In conclusion, we demonstrated that vascular inflammation and atherosclerosis are triggered by increased macrophage mitochondrial oxidative stress and dysfunction in hyperlipidemic conditions during aging. To combat age-related atherosclerosis and maintain plaque stability, improving the mitochondrial function of macrophages by inhibiting NOX4 activity and improving mitochondrial oxidative capacity could be advantageous.

## Data availability statement

The raw data supporting the conclusions of this article will be made available by the authors, without undue reservation.

## Ethics statement

The animal study was approved by University of Michigan Institutional Animal Care and Use Committee. The study was conducted in accordance with the local legislation and institutional requirements.

## Author contributions

AV: Conceptualization, Data curation, Formal analysis, Funding acquisition, Investigation, Methodology, Project administration, Resources, Software, Supervision, Validation, Visualization, Writing – original draft, Writing – review & editing. AL: Investigation, Methodology, Writing – original draft. TH: Investigation, Methodology, Writing – original draft. JL: Investigation, Methodology, Writing – original draft. JC: Investigation, Methodology, Writing – original draft. AA: Writing – review & editing. MR: Writing – review & editing. NM: Conceptualization, Data curation, Formal analysis, Funding acquisition, Investigation, Methodology, Project administration, Resources, Software, Supervision, Validation, Visualization, Writing – original draft, Writing – review & editing.
