# Supplementary material for: Mitochondrial dysfunction and metabolic reprogramming induce macrophage pro-inflammatory phenotype switch and atherosclerosis progression in aging
Source: Front Immunol. 2024 Jun 21;15:1410832. doi: 10.3389/fimmu.2024.1410832 (PMC11224442; doi:10.3389/fimmu.2024.1410832)
Supplement: Supplementary file 1 [file DataSheet_1.pdf]

Supplementary Material

Mitochondrial dysfunction and metabolic reprogramming induce macrophage pro-inflammatory phenotype switch and atherosclerosis progression in aging

Aleksandr E. Vendrov,[1] Andrey Lozhkin,[1] Takayuki Hayami,[1] Julia Levin,[1] Jamille Silveira Fernandes Chamon,[1] Ahmed Abdel-Latif,[1,2] Marschall S. Runge,[1] Nageswara R. Madamanchi[1]*

[1]Frankel Cardiovascular Center, Division of Cardiovascular Medicine, Department of Internal Medicine, University of Michigan, Ann Arbor, MI 48109, USA
[2]Ann Arbor VA Healthcare System, Ann Arbor, MI 48109, USA
*Correspondence: Nageswara R. Madamanchi madamanc@med.umich.edu

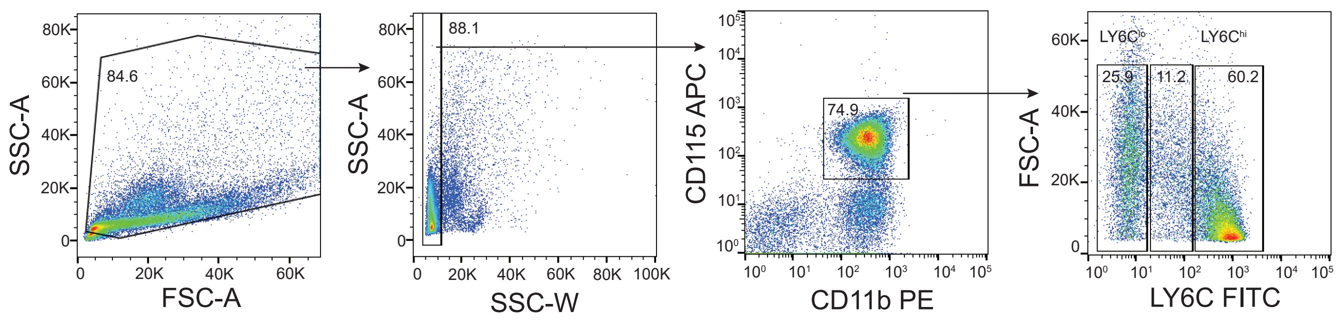

Supplementary Figure 1. Flow cytometry analysis of peripheral blood mononuclear cells. Gating strategy to quantify LY6C$^{hi/lo}$CD11b^{+}CD115^{+} monocytes.

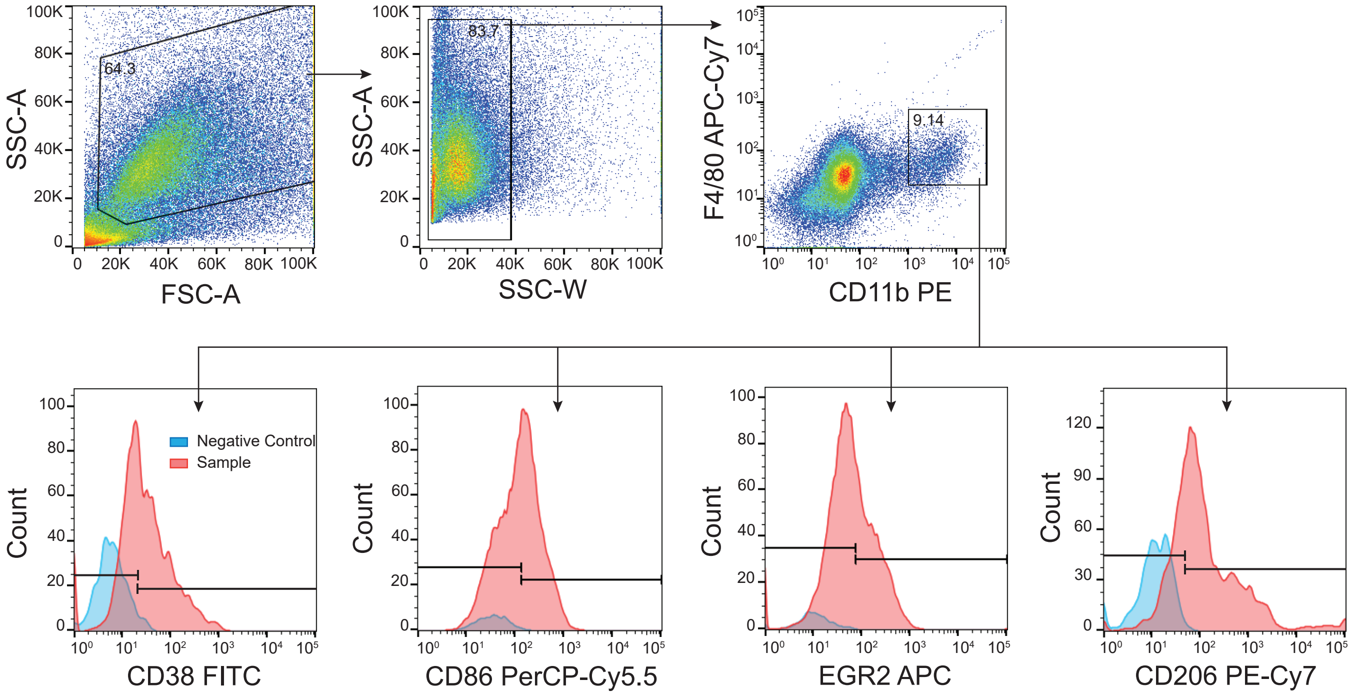

Supplementary Figure 3. Flow cytometry analysis of atherosclerotic plaque macrophages. Gating strategy to quantify $CD38^+$, $CD86^+$, $EGR2^+$, and $CD206^+$ $CD11b^+F4/80^+$ macrophages. The gates were set based on the signal from unstained cells (negative control) and single stain compensation beads (positive control). Representative overlay images showing negative control (blue) and sample signals (red).

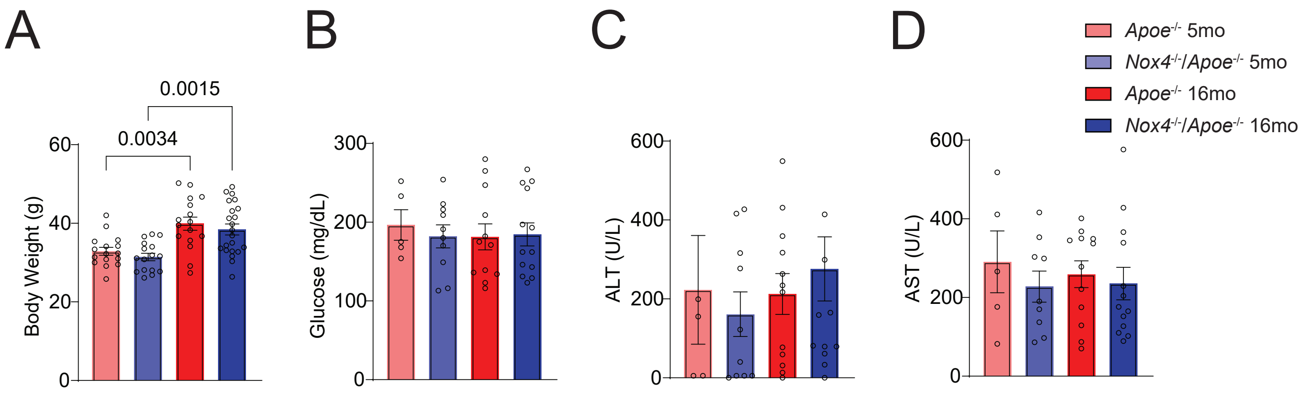

Supplementary Figure 3. Metabolic changes in young and aged *Apoe*$^{-/-}$ and *Nox4*$^{-/-}$/*Apoe*$^{-/-}$ mice. (A) Body weight in young (5-month-old) and aged (16-month-old) *Apoe*$^{-/-}$ and *Nox4*$^{-/-}$/*Apoe*$^{-/-}$ mice fed Western diet for 3 months (mean±SEM, n=16). (B) Plasma glucose levels (mean±SEM, n=10). (C) Plasma alanine aminotransferase (ALT) levels (mean±SEM, n=10). (D) Plasma aspartate transaminase (AST) levels (mean±SEM, n=10).

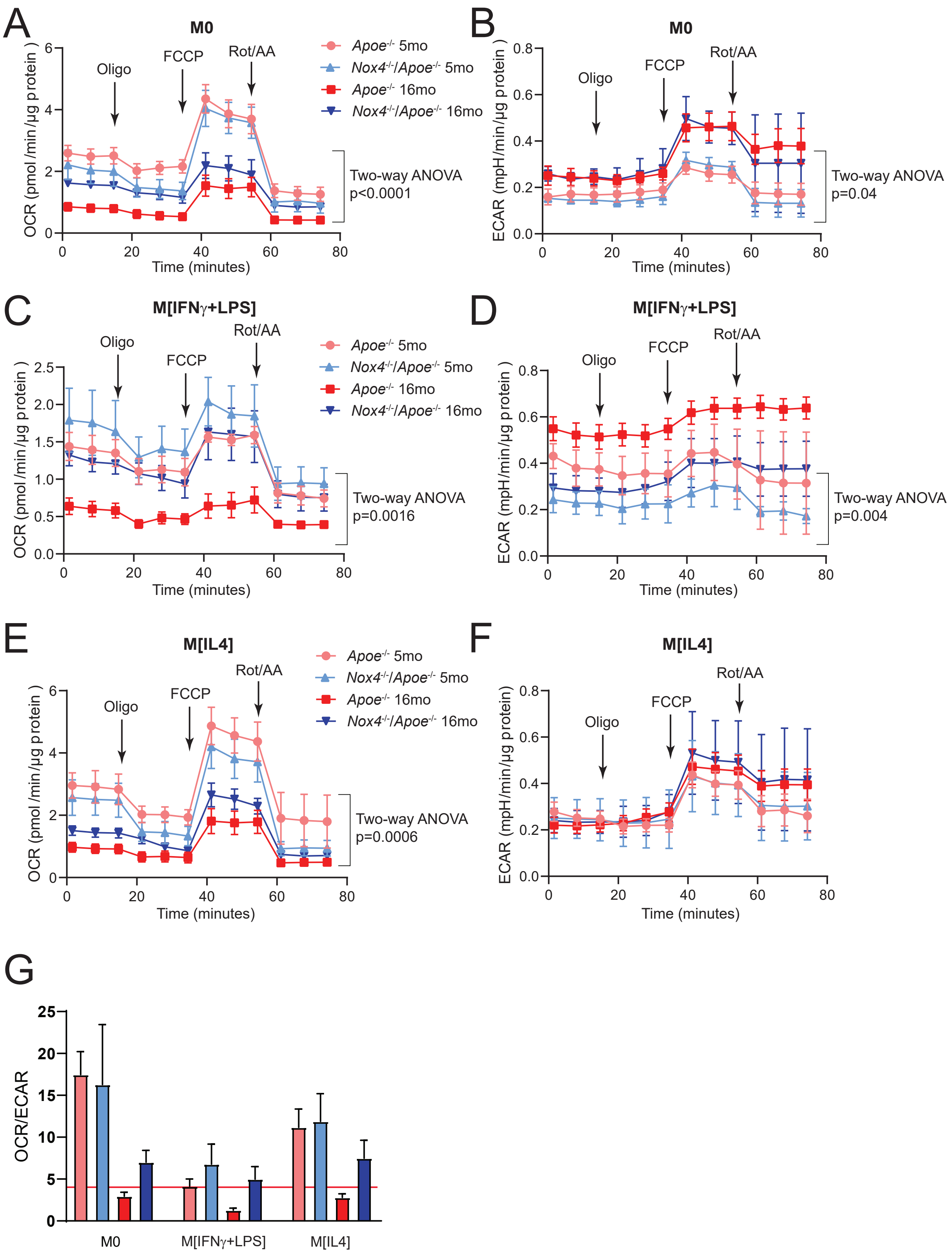
A
M0
Oligo
FCCP
Rot/AA
OCR (pmol /min /μg protein)
Time (minutes)
Apoe-/- 5mo
Nox4-/-/Apoe-/- 5mo
Apoe-/- 16mo
Nox4-/-/Apoe-/- 16mo
Two-way ANOVA
p<0.0001
B
M0
Oligo
FCCP
Rot/AA
ECAR (mpH/min/μg protein)
Time (minutes)
Two-way ANOVA
p=0.04
C
M[IFNγ+LPS]
Oligo
FCCP
Rot/AA
OCR (pmol /min /μg protein)
Time (minutes)
Apoe-/- 5mo
Nox4-/-/Apoe-/- 5mo
Apoe-/- 16mo
Nox4-/-/Apoe-/- 16mo
Two-way ANOVA
p=0.0016
D
M[IFNγ+LPS]
Oligo
FCCP
Rot/AA
ECAR (mpH/min/μg protein)
Time (minutes)
Two-way ANOVA
p=0.004
E
M[IL4]
Oligo
FCCP
Rot/AA
OCR (pmol /min /μg protein)
Time (minutes)
Apoe-/- 5mo
Nox4-/-/Apoe-/- 5mo
Apoe-/- 16mo
Nox4-/-/Apoe-/- 16mo
Two-way ANOVA
p=0.0006
F
M[IL4]
Oligo
FCCP
Rot/AA
ECAR (mpH/min/μg protein)
Time (minutes)
G
OCR/ECAR
M0
M[IFNγ+LPS]
M[IL4]

Supplementary Figure 4. Measurement of mitochondrial respiration and glycolysis in monocyte-derived macrophages from young and aged *Apoe*$^{-/-}$ and *Nox4*$^{-/-}$/*Apoe*$^{-/-}$ mice. (A) Oxygen consumption rate (OCR) was determined using Seahorse analyzer in M0 macrophages. Two-way repeated measures ANOVA test showed significant differences between young and aged macrophages ($p<0.0001$). (B) ECAR measurements in M0 macrophages. Two-way ANOVA test showed significant differences between young and aged macrophages ($p=0.0381$). (C) OCR measurements in M[IFNγ+LPS] macrophages. Two-way ANOVA test showed significant differences between young and aged *Apoe*$^{-/-}$ and *Nox4*$^{-/-}$/*Apoe*$^{-/-}$ macrophages ($p=0.0016$). (D) ECAR measurements in M[IFNγ+LPS] macrophages. Two-way ANOVA test showed significant differences between young and aged *Apoe*$^{-/-}$ and *Nox4*$^{-/-}$/*Apoe*$^{-/-}$ macrophages ($p=0.0047$). (E) OCR measurements in M[IL4] macrophages. Two-way ANOVA test showed significant differences between young and aged *Apoe*$^{-/-}$ and *Nox4*$^{-/-}$/*Apoe*$^{-/-}$ macrophages ($p=.0006$). (F) ECAR measurements in M[IL4] macrophages. Two-way ANOVA analysis did not show significant differences. (G) OCR/ECAR ratio in M0, M[IFNγ+LPS], and M[IL4] macrophages. OCR/ECAR=4 denoted by red line. The ratio below 4 suggests the preference for glycolysis for energy production.

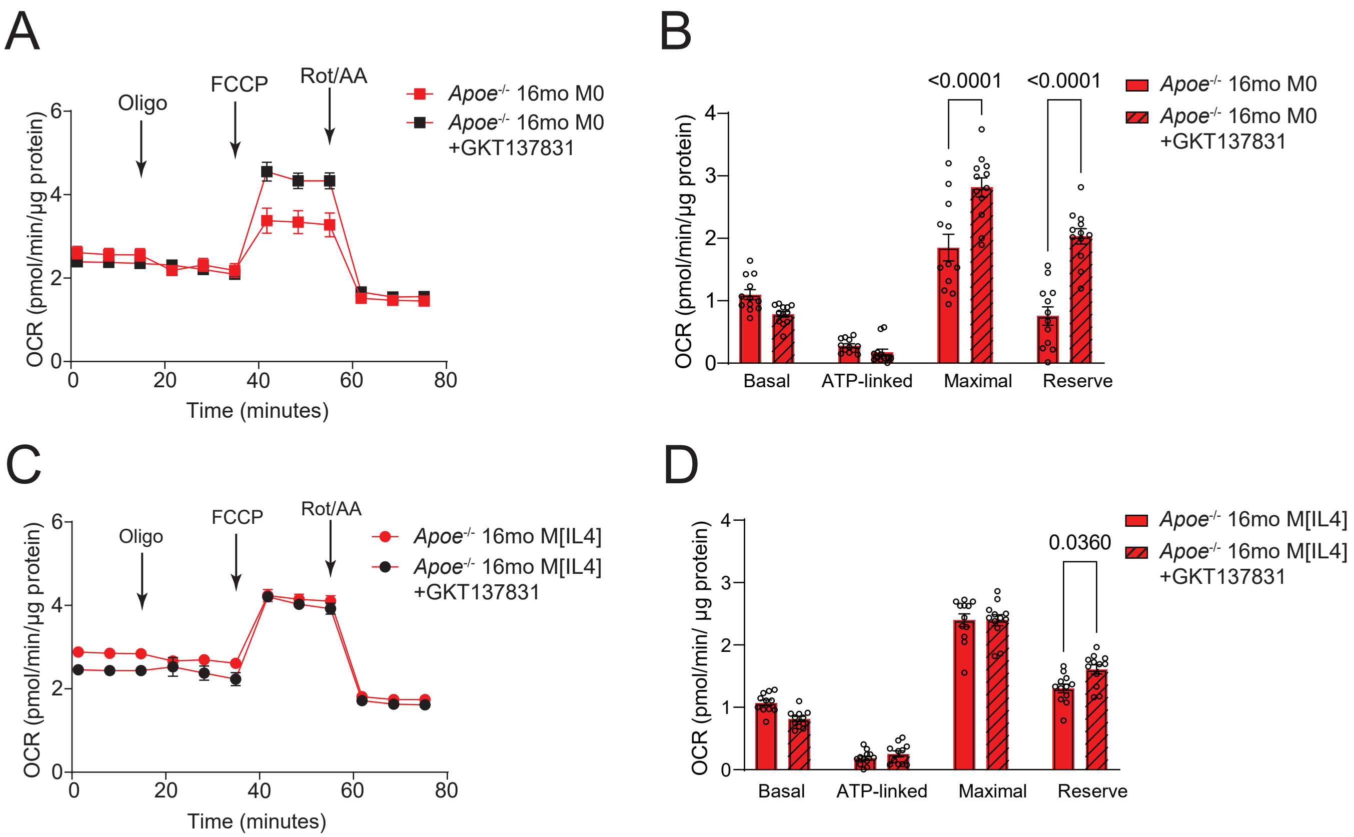

Supplementary Figure 5. Effect of GKT137831 treatment on mitochondrial respiration and bioenergetics parameters in monocyte-derived macrophages from aged *Apoe*$^{-/-}$ mice. (A-B) Oxygen consumption rate (OCR) measurements (A) and mitochondria bioenergetic parameters (B) in M0 macrophages. (C-D) Oxygen consumption rate (OCR) measurements (C) and mitochondria bioenergetic parameters (D) in M[IL4] macrophages.
